# Inferring human miRNA–disease associations via multiple kernel fusion on GCNII

**DOI:** 10.3389/fgene.2022.980497

**Published:** 2022-09-05

**Authors:** Shanghui Lu, Yong Liang, Le Li, Shuilin Liao, Dong Ouyang

**Affiliations:** ^1^ School of Computer Science and Engineering, Macau University of Science and Technology, Taipa, China; ^2^ School of Mathematics and Physics, Hechi University, Hechi, China; ^3^ Peng Cheng Laboratory, Shenzhen, China

**Keywords:** miRNA-disease associations, GCNII, dual laplacian regularized least squares, deep GCN, multiple kernel fusion

## Abstract

Increasing evidence shows that the occurrence of human complex diseases is closely related to the mutation and abnormal expression of microRNAs(miRNAs). MiRNAs have complex and fine regulatory mechanisms, which makes it a promising target for drug discovery and disease diagnosis. Therefore, predicting the potential miRNA-disease associations has practical significance. In this paper, we proposed an miRNA–disease association predicting method based on multiple kernel fusion on Graph Convolutional Network via Initial residual and Identity mapping (GCNII), called MKFGCNII. Firstly, we built a heterogeneous network of miRNAs and diseases to extract multi-layer features via GCNII. Secondly, multiple kernel fusion method was applied to weight fusion of embeddings at each layer. Finally, Dual Laplacian Regularized Least Squares was used to predict new miRNA–disease associations by the combined kernel in miRNA and disease spaces. Compared with the other methods, MKFGCNII obtained the highest AUC value of 0.9631. Code is available at https://github.com/cuntjx/bioInfo.

## 1 Introduction

An microRNA (abbreviated miRNA) is a small single-stranded non-coding RNA molecule (containing about 22 nucleotides) found in plants, animals and some viruses that functions in RNA silencing and post-transcriptional regulation of gene expression (David (2018); Qureshi et al. (2014)). The first miRNA was discovered in 1993 by a group led by Ambros and including Lee and Feinbaum (Lee R. C. et al. (1993)). In 2000, the second small RNA was characterized: let-7 RNA, which represses lin-41 to promote a later developmental transition in *C. elegans* (Reinhart et al. (2000)). The let-7 RNA was found to be conserved in many species, leading to the suggestion that let-7 RNA and additional “small temporal RNAs” might regulate the timing of development in diverse animals, including humans (Pasquinelli et al. (2000)). The dysfunction of miRNAs and their target mRNAs may result in various human diseases (Bandyopadhyay et al. (2010)). For instance, downregulation of miR-15 and miR-16 miRNAs also appears to be a feature of pituitary adenomas (Osada and Takahashi (2007)). Three miRNAs showed significantly more underexpression compared to the other downregulated miRNAs. These miRNAs are as follows: mir-127, mir-130a and mir-144 (Cahill et al. (2007)). The identification of miRNA-disease associations contributes to a better understanding of the relationship between miRNA and disease and the developing of new therapeutic drugs and therapeutic targeting miRNA (Brunetti et al. (2015); Chen et al. (2021)). Using biological experiments to identify the associations between miRNAs and diseases is time-consuming and expensive (Huang et al. (2019)). In the last few years many computational methods have been developed to explore the potential associations between miRNAs and diseases. According to different forecasting strategies, current methods can be divided into three categories: machine learning-based methods, information dissemination-based methods and similarity-based methods.

For machine learning-based methods, MLMDA (Zheng (2019)) was proposed to predict the associations of miRNAs and diseases. MLMDA extracts miRNA sequences by using a k-mer sparse matrix and incorporates the similarity of miRNAs and diseases. After being extracted by an autoencoder neural network, the features are fed into a random forest classifier to predict the associations between miRNAs and diseases. (Ji et al. (2020)). proposed a model called GraRep based on embedding-based heterogeneous information integration method which is adopted to learn the behavior information of miRNA and disease node in the network. And then, the random forest classifier is used to predict potential miRNA-disease associations. (Zhou et al. (2021)). proposed a model named DAEMKL for predicting miRNA-disease associations via deep autoencoder with multiple kernel learning. Sample imbalance is a major problem in this type of methods.

For information dissemination-based methods, (Chen et al. (2017a)), proposed a model called HAMDA which made use of the hybrid graph-based recommendation algorithm and extended previous recommendation algorithm by combing the usage of network structure, information propagation and adding more field-related information into the disease-miRNA association network. (Chen et al. (2018)). proposed a model named heterogeneous label propagation (HLPMDA), in which a heterogeneous label was propagated on multi-network and the model can calculate the strength of the data of associations which help to produce a better prediction. This type of methods relies on the connectivity of the network, and to increase connectivity, it is often necessary to add additional types of nodes and associations to the network.

Similarity-based methods are based on the hypothesis that similar functions of miRNAs are more likely to be related to the similar diseases. (Jiang et al. (2010)). created the method of forming both a functionally associated miRNA network and a human phenotypic one to find out whether the former ones are linked to phenotypically related diseases. At last, the potential miRNA-disease associations were predicted by the similarity score. WBSMDA (Chen et al. (2016)) calculated the within-score and between-score by integrating the similarity of miRNAs and diseases, and combined these scores to obtain the final scores for potential miRNA-disease association inference. In addition, it is also common practice to combine similarity with matrix transformation. (Xiao et al. (2018)). proposed a model called GRNMF, which integrated the disease semantic information and miRNA functional information to estimate disease similarity and miRNA similarity. And then, they used a graph to regularize non-negative matrix factorization framework to simultaneously identify potential associations for all diseases. (Li et al. (2021)). proposed a computational model called SCMFMDA, which based on similarity constrained matrix factorization for miRNA-disease associations prediction. These methods rely on the definition of similarity. In addition, similarity-based methods are also commonly used in microbe–disease associations studies. For example, (Yin et al. (2020)), proposed a model named NCPLP, which is based on network consistency projection and label propagation to infer potential microbe–disease associations. However, there is not any accepted evaluation method to account for the accuracy and reasonableness of similarity definitions.

In biological bipartite networks, Multiple Kernel Learning (MKL) (Gönen and Alpaydın (2011)) is a common method used to improve model performance. Firstly, MKL uses the multiple information of the samples to compute the multiple kernel matrix, and then obtains the optimal kernel matrix by fusing multiple kernel matrices. MKRMDA (Chen et al. (2017b)) based on MKL and Kronecker regularized least squares, which could automatically optimize the combination of multiple kernels for disease and miRNA, and achieved average AUCs of 0.8894 ± 0.0015 in five fold cross validation. (Qi et al. (2021)). presented a clustering method based on multiple kernel combination that can directly discover groupings in scRNA-seq data. MKLC-BiRW (Yan et al. (2019)) is proposed to predict new drug–target interactions by integrating diverse drug-related and target-related heterogeneous information. (Yang et al. (2022)). proposed a model based on Multiple Kernel fusion on Graph Convolutional Network with three layers, called MKGCN, for inferring novel microbe–drug associations. MKL can improve the prerformance of the model by combining a variety of information. Therefore, generally speaking, the more information is fed to the model, the easier it is to improve the predictive abilities of the model.

It is worth noting that researchers have begun to focus on identifying multiple types of miRNA-disease associations. (Chen et al. (2015)). was the first to study the problem. They developed a Restricted Boltzmann machine model (RBMMMDA) for multiple types of miRNA-disease association prediction. (Yu N. et al. (2022)). built a model named TFLP based on tensor factorization and label propagation. (Zhang et al. (2022)). proposed a signed graph neural network method (SGNNMD) to predict deregulation types of miRNA-disease associations. And WeightTDAIGN was proposed by (Ouyang et al. (2022)) later. All these models are capable of identifying multiple types of miRNA-disease associations, but the performance of these models is not yet as good as that of those designed to identify single potential type of miRNA–disease association.

As we all know, Graph Convolutional Networks (GCNs) (Kipf and Welling (2016)) generalize convolutional neural networks (CNNs) (LeCun and Bengio (1995)) to graph-structured data. GCN is being widely used in various biological problems (Yang et al. (2022); Han et al. (2019); Li et al. (2020); Zhao et al. (2021)). Most of the recent models based on GCN achieved their best performance with 2 or 3 layer models. Such shallow architectures limit their ability to extract information from high-order neighbors. However, stacking more layers and adding non-linearity tends to degrade the performance of these models. Such a phenomenon is called over-smoothing (Li et al. (2018)), which suggests that as the number of layers increases, the representations of the nodes in GCN are inclined to converge to a certain value and thus become indistinguishable. This over-smoothing phenomenon is neither a bug nor a special case, but an essential nature for GNNs. As mentioned before, MKL can improve the prerformance of the model by combining a variety of information. Therefore, it is advisable to combine MKL with a GNN model which can stack more layers to improve the model’s performance. (Chen et al. (2020)). proposed Graph Convolutional Network via Initial residual and Identity mapping (GCNII), a deep GCN model which can largely resolve the over-smoothing problem. In this paper, we propose a new model **M**ultiple **K**ernel **F**usion on **GCNII**(Chen et al. (2020)), called MKFGCNII, for predicting miRNA–disease associations. Firstly, we built a heterogeneous biological network including an miRNA network and a drug network. Secondly, we employed a multi-layer GCNII to extract the embedding features on each layer. Thirdly, we calculated the kernel matrix by the embedding features on each layer, and fused multiple kernel matrices based on a weighting method. Finally, Dual Graph Regularized Least Squares (DLapRLS) (Ding et al. (2020)) was used to predict new miRNA–disease associations by all the combined kernels. In the experiment, the best performance was achieved when the MKFGCNII model reached 16 layers. Under this condition, the performance of the MKFGCNII model under 5-fold cross-validation obtained the average area under the curve (AUC) of 0.9631 and area under the precision-recall (AUPR) of 0.9746. Furthermore, we also conducted case studies about esophageal neoplasms, lymphoma, and prostate neoplasms. The results showed that 48, 47, and 47 of the top 50 miRNAs related to these diseases were verified by dbDEMC and miR2Disease databases, respectively. Our experimental results demonstrated that the MKFGCNII model can be a usefull tool for helping researchers study miRNA-disease associations.

The main contributions of our article are as follows: 1) Our model applies the GCNII Network into Multiple Kernel fusion. 2) We apply deep layer GCNII to extract different structural information in the Heterogeneous graph. 3) Our model combines DLapRLS, MKL and GCNII and achieves good performance on the HMDD 2.0 (Li et al. (2014)) dataset.

## 2 Materials and methods

### 2.1 Human miRNA-disease associations database

The dataset used in this paper is HMDD v2.0 database which can be downloaded from https://www.cuilab.cn/hmdd (Li et al. (2014)). This dataset contains 495 miRNAs, 383 diseases, and 5,430 experimentally verified miRNA-disease associations. Inferring novel associations in human miRNA–disease network can be regarded as a kind of biological bipartite network prediction. In our experiment, we represented miRNAs and diseases as two different types of nodes in the network. The node set of **N**_*m*_ miRNAs is defined as $M=\left\{m_{1},\ldots ,m_{N_{m}}\right\}$ . Similarly, we described the node set of **N**_*d*_ diseases as $N=\left\{d_{1},\ldots ,d_{N_{d}}\right\}$ . An adjacency matrix $Y\in R^{N_{m}\times N_{d}}$ is created to store miRNA-disease associations. In this matrix, 495 rows represent the number of miRNAs, 383 columns represent the number of diseases. If miRNA *m*_*i*_ (1 ≤ *i* ≤ *N*_*m*_) is associated with disease *d*_*j*_ (1 ≤ *j* ≤ *N*_*d*_), **Y**_*ij*_ = 1, otherwise **Y**_*ij*_ = 0.

### 2.2 MiRNA functional similarity

(Wang et al. (2010)) proposed a model to calculate miRNAs functional similarity, which was based on the assumption that miRNAs with similar functions are often connected with similar diseases and vice versa. Based on (Wang et al. (2010))’s previous work, we can download the miRNA functional similarity data from https://www.cuilab.cn/files/images/cuilab/misim.zip directly. In this paper, we constructed a matrix $MFS\in R^{N_{m}\times N_{m}}$ to describe the functional similarity between miRNAs, where element **MFS**(*m*_*i*_, *m*_*j*_) represents the functional similarity between miRNA *m*_*i*_ and *m*_*j*_.

### 2.3 Disease semantic similarity

Based on (Xuan et al. (2013)) and (Schriml et al. (2012))’s study, firstly, we got the relationships between different diseases from the medical subject headings (MeSH) database (https://www.ncbi.nlm.nih.gov/). Then, we constructed the disease semantic similarity networks by using Disease Ontology information and calculated disease semantic similarity. Every disease can be represented by a directed acyclic graph (DAG) in the MeSH database. *DAG* (*d*_*i*_) = (*d*_*i*_, *T* (*d*_*i*_), *E* (*d*_*i*_)) represents a directed acyclic graph of disease *d*_*i*_, which contains disease *d*_*i*_, its ancestor nodes *T* (*d*_*i*_), and the set of directly connected edges *E* (*d*_*i*_) from the ancestor nodes to node *T* (*d*_*i*_). Then, the semantic contribution value of disease *d*_*k*_ to *d*_*i*_ can be calculated as follows:

$$SC1_{d_{i}}\left(d_{k}\right)=\left\{\begin{array}{cc} 1, & \text{if }d_{k}=d_{i}\\ \max\left\{\Delta \times SC1_{d_{i}}\left(d_{k^{\prime }}\right)\right\}, & \text{other} \end{array}\right..$$ (1)

where *d*_*k*’_ denotes the children node of *d*_*k*_, Δ denotes the contributing factor of semantic decay, which was set to 0.5 according to Xuan et al. (2013). The contributing factor of disease *d*_*i*_ to itself was set to 1. From Eq. 1 we know that if the distance from disease *d*_*k*_ to disease *d*_*i*_ increases, the semantic contribution factor will decrease. Then, the semantic value of disease *d*_*i*_ can be calculated by:

$$SV1\left(d_{i}\right)=\underset{d_{k}\in T\left(d_{i}\right)}{\sum }SC1_{d_{i}}\left(d_{k}\right).$$ (2)

According to the assumption that the more DAGs are shared between diseases, the more similar they are. The disease semantic similarity **DS1** (*d*_*i*_, *d*_*j*_) between disease *d*_*i*_ and *d*_*j*_ can be calculated by utilizing the following formula:

$$DS1\left(d_{i},d_{j}\right)=\frac{\sum_{d_{k}\in T\left(d_{i}\right)\cap T\left(d_{j}\right)}\left(SC1_{d_{i}}\left(d_{k}\right)+SC1_{d_{j}}\left(d_{k}\right)\right)}{SV1\left(d_{i}\right)+SV1\left(d_{j}\right)}.$$ (3)

In order to predict miRNA-disease associations, (Pasquier and Gardès (2016)), investigated the hypothesis that information attached to miRNAs and diseases can be revealed by distributional semantics to calculate disease semantic similarity. So, the distributional information on miRNAs and diseases can be represented in a high-dimensional vector space. In this way, every appearance of diseases in the same layer of DAG can be taken into account. The semantic contribution value of disease *d*_*k*_ to *d*_*i*_ can be calculated as follows:

$$SC2_{d_{i}}\left(d_{k}\right)=-\log\left(\frac{num\left(DAGs\left(d_{k}\right)\right)}{N_{d}}\right).$$ (4)

Then, the semantic value of disease *d*_*i*_ is calculated by Eq. 5 and the disease semantic similarity **DS2** (*d*_*i*_, *d*_*j*_) between disease *d*_*i*_ and *d*_*j*_ is calculated by Eq. 6 as follows:

$$SV2\left(d_{i}\right)=\underset{d_{k}\in T\left(d_{i}\right)}{\sum }SC2_{d_{i}}\left(d_{k}\right),$$ (5)

$$DS2\left(d_{i},d_{j}\right)=\frac{\sum_{d_{k}\in T\left(d_{i}\right)\cap T\left(d_{j}\right)}\left(SC2_{d_{i}}\left(d_{k}\right)+SC2_{d_{j}}\left(d_{k}\right)\right)}{SV2\left(d_{i}\right)+SV2\left(d_{j}\right)}.$$ (6)

We integrated *DS*1 and *DS*2 together as the final disease semantic similarity for a better disease semantic similarity. The final disease semantic similarity is defined as follows:

$$DSS\left(d_{i},d_{j}\right)=\frac{DS1\left(d_{i},d_{j}\right)+DS2\left(d_{i},d_{j}\right)}{2}.$$ (7)

### 2.4 Gaussian interaction profile kernel similarity for diseases and miRNAs

To obtain topological information of miRNAs and diseases in relational graphs, we can calculate the Gaussian interaction profile kernel similarity for miRNAs and diseases by using miRNA-disease association network (Chen et al. (2016)). Firstly, based on assumptions that similar miRNAs are more likely to be associated with similar diseases, we utilized a binary vector **BI**(*m*_*i*_), which is the *ith* row of matrix **Y**, representing the associations between miRNA *m*_*i*_ and all diseases. Then, the Gaussian interaction profile kernel similarity for miRNAs **MGS** (*m*_*i*_, *m*_*j*_) between miRNA *m*_*i*_ and *m*_*j*_ can be calculated as below:

$$MGS\left(m_{i},m_{j}\right)=exp\left(\gamma_{m}\|BI\left(m_{i}\right)-BI\left(m_{j}\right){\|}^{2}\right),$$ (8)

$$\gamma_{m}=\alpha_{m}/\left(\frac{1}{N_{m}}\overset{N_{m}}{\underset{i=1}{\sum }}\|BI\left(m_{i}\right)\left.\right){\|}^{2}\right).$$ (9)

Here, *α*_*m*_ has been set to 1 referring to Chen et al. (2016)’s studies. Taking the same approach, we can calculate the Gaussian interaction profile of diseases **MGS** (*m*_*i*_, *m*_*j*_) between diseases *d*_*i*_ and *d*_*j*_ as follows:

$$DGS\left(d_{i},d_{j}\right)=exp\left(\gamma_{d}\|BI\left(d_{i}\right)-BI\left(d_{j}\right){\|}^{2}\right),$$ (10)

$$\gamma_{d}=\alpha_{d}/\left(\frac{1}{N_{d}}\overset{N_{d}}{\underset{i=1}{\sum }}\|BI\left(d_{i}\right)\left.\right){\|}^{2}\right).$$ (11)

Here, a binary vector **BI**(*d*_*i*_), which is the *ith* column of matrix **Y**, represents the associations between disease *d*_*i*_ and all miRNAs. *α*_*d*_ has been set to 1 referring to (Chen et al. (2016))’s studies.

### 2.5 Integrated similarity for miRNAs and diseases

By integrating the calculations above, we can get the integrated similarity for miRNAs **IM**_0_ (*m*_*i*_, *m*_*j*_) between miRNA *m*_*i*_ and *m*_*j*_ as Eq. 12, and the integrated similarity for diseases **ID**_0_ (*d*_*i*_, *d*_*j*_) between disease *d*_*i*_ and *d*_*i*_ as Eq. 13.

$${IM}_{0}\left(m_{i},m_{j}\right)=\left\{\begin{array}{cc} MFS\left(m_{i},m_{j}\right), & \text{if }MFS\left(m_{i},m_{j}\right)\text{ exits}\\ MGS\left(m_{i},m_{j}\right), & \text{otherwise} \end{array}\right.,$$ (12)

$${ID}_{0}\left(m_{i},m_{j}\right)=\left\{\begin{array}{cc} DSS\left(d_{i},d_{j}\right), & \text{if }DSS\left(d_{i},d_{j}\right)\text{ exits}\\ DGS\left(d_{i},d_{j}\right), & \text{otherwise} \end{array}\right..$$ (13)

### 2.6 Heterogeneous network

Inspired by (Yang et al. (2022)), we built a heterogeneous biological network including an miRNA network **IM**_0_, a disease network **ID**_0_, and an association network between miRNAs and diseases. Finally, we constructed the heterogeneous network defined by the adjacency matrix $A\in R^{\left(N_{m}+N_{d}\right)\times \left(N_{m}+N_{d}\right)}$ :

$$A=\left[\begin{array}{cc} {IM}_{0} & Y\\ Y^{T} & {ID}_{0} \end{array}\right].$$ (14)

### 2.7 Deep graph convolutional network

As we know, Graph Convolutional Network (GCN) is a neural network that can learn low dimensional representation. However, stacking more layers and adding non-linearity will cause GCN to appear *over-smoothing*. Therefore, we applied the GCNII (Chen et al. (2020)) model which is a deep model that can effectively extract the embedding from the graph and partially solve the problem of *over-smoothing* to extract the embedding of heterogeneous graph on each layer.

Specifically, given a heterogeneous network adjacency matrix *A* as defined above, the GCNII model of the heterogeneous network can be defined as follows:

$$H^{\left(l+1\right)}=\sigma \left(\left(1-\alpha_{l}\right)\tilde{P}H^{\left(l\right)}+\alpha_{l}H^{\left(0\right)}\right)\times \left(\left(1-\beta_{l}\right)I_{n}+\beta_{l}W^{\left(l\right)}\right).$$ (15)

where **H** ^(*l*)^ is the *l*th layer embedding of nodes, where *l* = 1, …, *L*;*α*_*l*_, *β*_*l*_ are hyperparameters, we set *α*_*l*_ = *α* in our method, $\beta_{l}=\log\left(\frac{\lambda }{l}+1\right)\approx \frac{\lambda }{l}$ , *λ* is hyperparameter. $\tilde{P}={\tilde{D}}^{-1/2}\tilde{A}{\tilde{D}}^{-1/2}$ , $\tilde{P}={\tilde{D}}^{-1/2}\tilde{A}{\tilde{D}}^{-1/2}={\left(D+I_{n}\right)}^{-1/2}\left(A+I_{n}\right){\left(D+I_{n}\right)}^{-1/2}$ , **D** the diagonal degree matrix of **A**, $W^{\left(l\right)}\in R^{\left(N_{m}+N_{d}\right)\times \left(k_{i}\right)}$ is a learnable weight matrix for the *l*th neural network layer and *k*_*i*_ is the dimensionality of embeddings of *lth* layer GCNII, *σ*(⋅) is a non-linear activation function.

In our study, we employed ReLU(Rectified Linear Unit) as the non-linear activation function. We constructed the initial embedding for the first layer **H** ^(0)^, **H** ^(1)^ and the last layer **H** ^(*L*+1)^ as follows:

$$H^{\left(0\right)}=\left[\begin{array}{cc} 0 & Y\\ Y^{T} & 0 \end{array}\right],$$ (16)

$$H^{\left(1\right)}=GAT\left(H^{\left(0\right)}\right),$$ (17)

$$H^{\left(L+1\right)}=W^{\left(L+1\right)}H^{\left(L\right)}+b_{\left(L+1\right)}.$$ (18)

where **GAT**(Veličković et al. (2017)) represents a two-layers GAT model, **W** ^(*L*+1)^, **b**_(*L*+1)_ are the weight matrix and bias of the fully connected layer, respectively.

### 2.8 Multi-kernal fusion

We can extract multiple embeddings for multi-layer GCNII model, which represents information of different graph structures. Specifically, **H** ^0^ represents the initial features of nodes in the heterogeneous graph, and **H** ^*l*+1^ (*l* = 1, *…*, *L*) aggregates the *l*-order neighbor information of nodes and original features according to the weight parameter *α*_*l*_ in Eq. 15. According to Eq. 15, we know that the embedding of each layer will be accompanied by the initial embedding. Thus, the problem of *over-smoothing* can be partially solved by controlling the hyperparameter *α*_*l*_, which means that the information aggregation of each layer can effectively avoid the phenomenon of homogenization, facilitating the execution of downstream tasks. Therefore, the embedding information on each layer can effectively represent different information. Thus, it is reasonable for us to perform multi-kernal fusion on these, and then use the fused information to make predictions.

For the embedding of *l*th layer **H** ^(*l*)^(*l* = 1, *…*, *L*), we can divide **H** ^(*l*)^ into two parts. The first *N*_*m*_ lines are used as miRNA embeddings and expressed as $H_{m}^{\left(l\right)}$ , and the last *N*_*d*_ lines are used as disease embeddings; then,the embedding of each layer can be represented as $H^{\left(l\right)}=\left[\begin{array}{c} H_{m}^{\left(l\right)}\\ H_{d}^{\left(l\right)} \end{array}\right]\in R^{\left(N_{m}+N_{d}\right)\times k_{i}},H_{m}^{\left(l\right)}\in R^{N_{m}\times k_{l}},\text{and }H_{d}^{\left(l\right)}\in R^{N_{d}\times k_{l}}$ .Finally, we used $H_{m}^{\left(l\right)}$ , $H_{d}^{\left(l\right)}$ and Gaussian interaction profile kernal similarity function to calculate the miRNA and disease kernel matrices on *l*th layer as follows:

$${IM}_{l}\left(i,j\right)=\exp\left(-\gamma_{l}\|H_{m}^{\left(l\right)}\left(i\right)-H_{m}^{\left(j\right)}{\|}^{2}\right),$$ (19)

$${ID}_{l}\left(i,j\right)=\exp\left(-\gamma_{l}\|H_{d}^{\left(l\right)}\left(i\right)-H_{d}^{\left(j\right)}{\|}^{2}\right).$$ (20)

where ${IM}_{l}\in R^{N_{m}\times N_{m}}$ , ${ID}_{l}\in R^{N_{d}\times N_{d}}$ , $H_{m}^{\left(l\right)}\left(i\right)$ and $H_{d}^{\left(l\right)}\left(i\right)$ represent the *i*th row in the *l*th layer miRNA and disease embeddings, i.e. the *i*th row of $H_{m}^{\left(l\right)}$ and $H_{d}^{\left(l\right)}$ , respectively; *γ*_*l*_ denotes the corresponding bandwidth, we set *γ*_*l*_ = *γ*, *l* = 1, …, *L*.

In order to make full use of the information to improve the performance of predicting miRNA–disease associations, we integrated all the kernels above with multiple kernel fusion, then adopted the weighted sum method to combine all kernel matrices. The combined kernel can be defined as follows:

$$IM=\overset{L+1}{\underset{i=0}{\sum }}\omega_{i}^{m}{IM}_{i},$$ (21)

$$ID=\overset{L+1}{\underset{i=0}{\sum }}\omega_{i}^{d}{ID}_{i}.$$ (22)

where $IM\in R^{N_{m}\times N_{m}}$ , $ID\in R^{N_{d}\times N_{d}}$ , $\omega_{i}^{m}=\frac{\mu }{n+{\left(i+1\right)}^{\theta }}$ , and $\omega_{i}^{d}=\frac{\mu }{n+{\left(i+1\right)}^{\theta }}$ are the corresponding weight of miRNA kernels and disease kernals, respectively; *n* is the number of hidden layers of GCNII. *μ* and *θ* are hyperparameters. Here, we set $\mu =\frac{n}{2}$ .

### 2.9 Dual Laplacian regularized least squares model

Inspired by (Ding et al. (2020)) and (Yang et al. (2022)), we adopted the Dual Laplacian Regularized Least Squares (DLapRLS) method to predict miRNA–disease associations. DLapRLS can avoids overfitting by adding graph regularization. Thus, the loss function can be defined as follows:

$$\begin{array}{ll} \text{min}J= & \|IM\alpha_{m}+{\left(ID\alpha_{d}\right)}^{T}-2Y_{\mathrm{train}}{\|}_{F}^{2}\\ & +\phi_{m}tr\left(\alpha_{m}^{T}L_{m}\alpha_{m}\right)+\phi_{d}tr\left(\alpha_{d}^{T}L_{d}\alpha_{d}\right). \end{array}$$ (23)

where ‖ ⋅‖_*F*_ is the Frobenius norm, $Y_{\mathrm{train}}\in R^{N_{m}\times N_{d}}$ is the adjacency matrix for miRNA–disease associations in the training set; ***α***_*m*_ and $\alpha_{d}^{T}\in R^{N_{m}\times N_{d}}$ are learnable matrices; $L_{m}\in R^{N_{m}\times N_{m}}$ and $L_{d}\in R^{N_{d}\times N_{d}}$ are the normalized Laplacian matrices, as follows:

$$L_{m}=D_{m}^{-1/2}\Delta_{m}D_{m}^{-1/2},\Delta_{m}=D_{m}-{IM}_{m},$$ (24)

$$L_{d}=D_{d}^{-1/2}\Delta_{d}D_{d}^{-1/2},\Delta_{d}=D_{d}-{ID}_{d}.$$ (25)

where $D_{m}=\sum_{i=1}^{N_{m}}IM$ and $D_{d}=\sum_{i=1}^{N_{d}}ID$ are diagonal degree matrix. Finally, we can obtain the prediction $\hat{F}$ for miRNA–disease associations from **IM** and **ID** as follows:

$$\hat{F}=\frac{IM\alpha_{m}+{\left(ID\alpha_{d}\right)}^{T}}{2}.$$ (26)

### 2.10 Training

We used Adam (Da (2014)) to update the parameters of GCNII, and then got the iterative function directly by calculating the partial derivatives for the parameters of DLapRLS. We first assume that ***α***_*d*_ is a constant matrix when we optimize ***α***_*m*_. Thus, the partial derivative of the loss function Eq. 23 with respect to ***α***_*m*_ can be calculated as follows:

$$\frac{\partial J}{\partial \alpha_{m}}=2IM\left(IM\alpha_{m}+{\left(ID\alpha_{d}\right)}^{T}-2Y_{\mathrm{train}}\right)+2\phi_{m}L_{m}\alpha_{m}$$ (27)

By letting $\frac{\partial J}{\partial \alpha_{m}}=0$ , ***α***_*m*_ can be obtain as follows:

$$\begin{array}{ll} & \left(IMIM+\phi_{m}L_{m}\right)\alpha_{m}=IM\left[2Y_{\mathrm{train}}-\alpha_{d}^{T}{ID}^{T}\right],\\ & \alpha_{m}={\left(IMIM+\phi_{m}L_{m}\right)}^{-1}IM\left[2Y_{\mathrm{train}}-\alpha_{d}^{T}{ID}^{T}\right]. \end{array}$$ (28)

Similarly, the partial derivative of the loss function Eq. 23 with respect to ***α***_*d*_ can be calculate as follows:

$$\frac{\partial J}{\partial \alpha_{d}}=2ID\left(ID\alpha_{d}+{\left(IM\alpha_{m}\right)}^{T}-2Y_{\mathrm{train}}^{T}\right)+2\phi_{d}L_{d}\alpha_{d}.$$ (29)

Similar to above, by letting $\frac{\partial J}{\partial \alpha_{d}}=0$ , ***α***_*d*_ can be obtain as follows:

$$\begin{array}{ll} & \left(IDID+\phi_{d}L_{d}\right)\alpha_{d}=ID\left[2Y_{\mathrm{train}}^{T}-\alpha_{m}^{T}{IM}^{T}\right],\\ & \alpha_{d}={\left(IDID+\phi_{d}L_{d}\right)}^{-1}ID\left[2Y_{\mathrm{train}}^{T}-\alpha_{m}^{T}{IM}^{T}\right]. \end{array}$$ (30)

**FIGURE 1:**
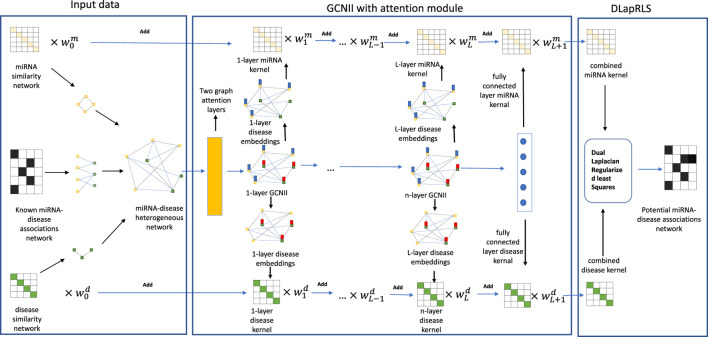
The overview of our proposed method.

We randomly initialized all the trainable parameters at the beginning of our model training, and then calculated ***α***_*m*_ and ***α***_*d*_ by Eq. 29 and Eq. 30 directly in each iteration, other parameters were optimized by Adam. The flowchart of our proposed method is shown in Figure 1. And, the overview of our model is shown in Table 1. As we all know, the imbalance of positive and negative samples will lead to a bias towards broad categories, which will lead to overfitting of the model (Li et al. (2022)). We took the experimentally verified miRNA-disease associations as positive samples, and the unknown miRNA-disease associations as negative samples as (Li et al. (2022)) did. And then, we randomly selected the same number of negative samples from all the unknown miRNA-disease associations. In this way, we selected a total of 10,860 samples.

**TABLE 1:** Algorithm 1 Algorithm of our proposed method.

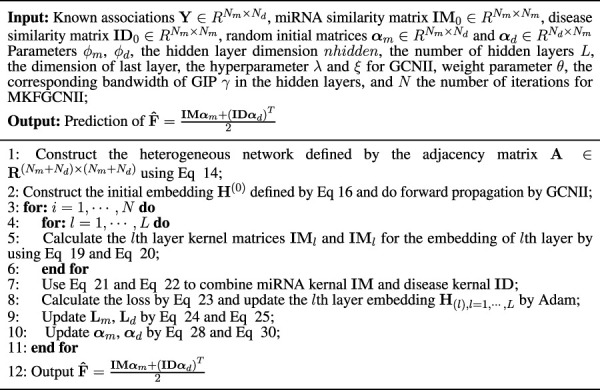

## 3 Result

### 3.1 Implementation details and performance evaluation

Our model was implemented based on PyTorch and PyG. In this experiment, we applied 5-fold cross-validation to evaluate the performance of our model, and we set training epochs to 15, the learning rate to 0.001, the weight decay of GCNII’s convolutional and fully connected layers to 0.001 and 0.0005, respectively; set the number of hidden layers to 16, the dimension hidden layers to 256, $\phi_{1}=\phi_{1}=\frac{1}{512}$ , dropout to 0.5, hyperparameters *ξ* = 0.5, *λ* = 2, $\theta =0.1,\gamma =\frac{1}{128}$ , respectively.

We drew tables to show the effect of the model. In Table 2, we can see that MKFGCNII achieves average Acc. of 92.59,Prec. of 92.91%, Recall of 91.57%, F1 score of 92.94%, AUC of 96.42%, and 97.35% with standard deviations of 0.24, 0.72, 0.49, 0.54, 0.25, and 0.22%, respectively.

**TABLE 2:** Five-fold cross-validation results performed by MKFGCNII based on HMDD v.2.0.

Testing set	Acc.(%)	Prec. (%)	Recall (%)	F1 score (%)	AUC (%)	AUPR (%)
1	92.27	91.78	93.27	92.52	96.67	97.44
2	92.77	93.15	92.73	92.94	96.56	97.51
3	92.82	93.78	92.10	92.93	96.15	97.18
4	92.68	92.95	92.70	93.83	96.59	97.58
5	92.40	92.88	92.13	92.50	96.15	97.06
Average	92.59 ± 0.24	92.91 ± 0.72	92.57 ± 0.49	92.94 ± 0.54	96.42 ± 0.25	97.35 ± 0.22

### 3.2 Compare with other latest methods

In order to evaluate the performance of our model in predicting the miRNAs-diseases associations, we compared the performance of the MKFGCNII model with six other latest models: DBMDA (Zheng et al. (2020)), CEMDA (Liu et al. (2021)), MDPBMP(Yu L. et al. (2022)), NIMCGCN(Li et al. (2020)), M2GMDA (Zhang et al. (2020)), MSHGATMDA (Wang et al. (2022)) and HGANMDA (Li et al. (2022)). We used the 5-fold cross-validation method on the same dataset HMDD v.2.0. as they did. The AUC values of the six models are shown in Table 3 and are 91.29, 92.03, 92.14, 92.91, 93.23, 93.45 and 93.74%, respectively. Our MKFGCNII obtained the highest AUC value of 96.42%. From Table 3, we can see that compared with the six models, our MKFGCNII model has the highest AUC value and it is 2.68% higher than the second highest HGANMDA model. There are two main possible reasons. The first is that DLapRLS has a good effect on predicting the relationship between two objects. And the second is that the depth of the model is deep enough to enable the model to fully extract various information for relationship prediction. In fact, in this experiment, our model had a total of 19 layers, 16 hidden layers are graph convolution layers, two graph attention layers are added between the input layer and the hidden layer, and a fully connected layer is added between the hidden layer and the output layer.

**TABLE 3:** The comparison results of MKFGCNII model with other latest models according to 5-fold cross-validation on HMDD v.2.0 dataset.

Method	AUC(%)
DBMDA (Zheng et al. (2020))	91.29
CEMDA (Liu et al. (2021))	92.03
MDPBMP(Yu et al. (2022a))	92.14
NIMCGCN(Li et al. (2020))	92.91
M2GMDA (Zhang et al. (2020))	93.23
MSHGATMDA (Wang et al. (2022))	93.45
HGANMDA (Li et al. (2022))	93.74
MKFGCNII(our)	**96.42**

Bold represents the maximum value.

### 3.3 Influence of hidden layers

In this experiment, we observed the effect of model depth on improving model performance by adjusting the number of hidden layers. The hidden layers were set to 2, 4, and 8 respectively. As mentioned above, a graph attention layer was added between the input layer and the hidden layer, and a fully connected layer was added between the hidden layer and the output layer. The comparison results are shown in Table 4. All experiments were performed with 5-fold cross-validation and trained with the same epoch. Finally, the average value of each evaluation was used as comparison. From Table 4, we can see that as the number of hidden layers increases, the model performance gets better, and the model performance has stabilized when the number of hidden layer reaches 8, which demonstrates the impact of model depth on model performance. It also proved that the GCNII module can not only solve the over-smoothing problem to a large extent, but also improve the model performance by increasing the number of hidden layers, which makes the performance of the MKFGCNII model better than the other models.

**TABLE 4:** Influence of hidden layers.

The number of hidden layers	Acc (%)	Prec. (%)	Recall (%)	F1 score (%)	AUC (%)	AUPR (%)
2	82.78	85.31	80.29	82.63	88.72	91.30
4	89.66	90.87	88.81	89.78	94.19	95.55
8	92.16	92.55	92.12	92.33	95.67	96.85
16	92.58	92.91	92.57	92.94	96.42	97.35

### 3.4 Other parameters evaluation

**FIGURE 2:**
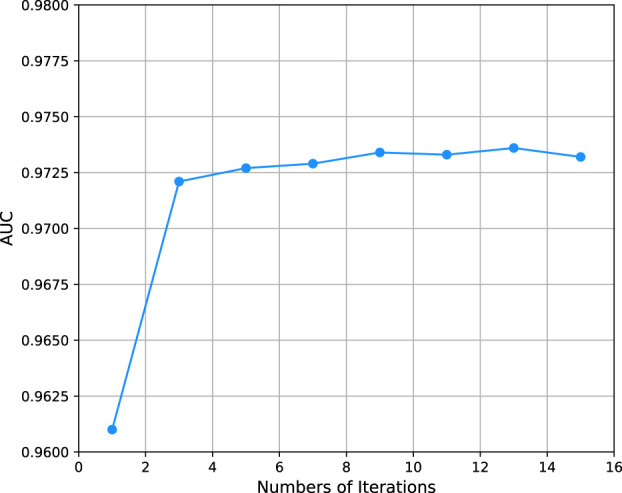
AUPR of models with different iterations.

In this experiment, we set the number of hidden layer to 16 and investigated the effect of other parameters of the model on model performance. Firstly, we evaluated the effect of iterations N which controls the times of updates of learnable parameters. From Figure 2A we can see that the AUPR values under different numbers of iterations. It shows that the AUPR values tends to stabilize when the number of iterations is 5. Thus, we evaluated the remaining parameters by iterating 10 times under 5-fold cross-validation.

**FIGURE 3:**
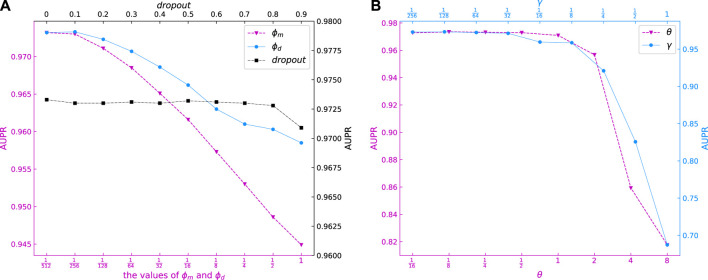
AUPR of models with different *dropout*, *ϕ*_*m*_, *ϕ*_*d*_, *γ* and *θ*.

The *ϕ*_*m*_ and *ϕ*_*d*_ represent the weights of graph regular terms in DLapRLS, and are important parameters of our model. Ten candidate values of {2^–9^, 2^–8^, *…*, 1} were selected for *ϕ*_*m*_ and *ϕ*_*d*_. Figure 3A shows the AUPR values for different *ϕ*_*m*_ and *ϕ*_*d*_ models. It can be seen that when *ϕ*_*m*_ and *ϕ*_*d*_ are small, the AUPR values higher. Our model obtains best AUPR with *ϕ*_*m*_ = 2^–9^ and *ϕ*_*d*_ = 2^–8^, respectively.

Different *θ* will generate different weights of miRNA and disease kernels. From Figure 3B we can see that the AUPR of our model is stable between $\frac{1}{16}$ and 1, then rapidly declines between 1 and 8. Thus, we set *θ* = 0.1 for our model. Different *γ* will generate different miRNA and disease kernels, which will affect the model performance. Figure 3B shows the effect of changes in *γ* on the AUPR of our model. It can be observed that AUPR gradually increases as *γ* decreases which means that smaller *γ* has a better effect on the predictive performance. Therefore, we set $\gamma =\frac{1}{128}$ for our model.

**FIGURE 4:**
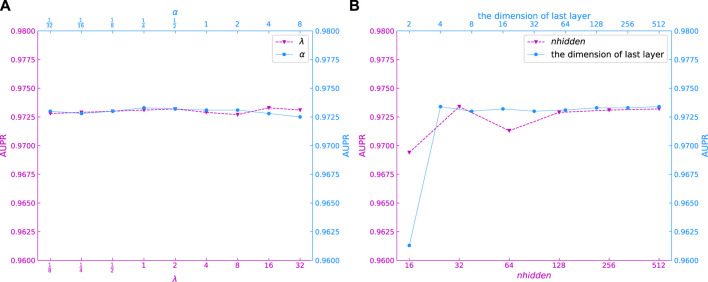
AUPR of models with different *α*, *λ*, *nhidden* and the dimension of last layer.

*λ* and *α* are the hyperparameters of module GCNII. Setting the hyperparameter of *λ* in GCNII module is to ensure the decay of the weight matrix adaptively increases when we stack more layers in GCNII module (Chen et al. (2020)). And, *α* means that the final representation of each node retains at least a proportion of *α* from the input layer, no matter how many layers we stack in module GCNII. It can be seen in Figure 4D that the AUPR of our model is stable when *λ* and *α* change between $\frac{1}{8}$ to 32 and between $\frac{1}{32}$ to 8. We set 2 and $\frac{1}{2}$ for *λ* and *α* for our model, respectively.

In the GCNII module of this experiment, the input features pass through a layer of GAT for inductive learning first and then enter the hidden layer. After passing through the 16 hidden layers, the output features are output through a layer of full connection, which means that in this experiment, the GCNII module contains two layers of full connection and 16 layers of graph convolution. Each graph convolutional layer has the same dimension. We used *nhidden* and *the dimension of last layer* denote the dimensions of the hidden layer vector and the output layer vector, respectively. 4(e) show the AUPR values for different *nhidden* and *the dimension of last layer* models. It can be seen that the values of AUPR is relatively high when *nhidden* takes 32, 128, 256 and 512, and the AUPR values become stable when *the dimension of last layer* is greater than 4. So, we set *nhidden* and *the dimension of last layer* to 256 and 64, respectively.

Finally, we evaluated the *dropout* values of our model. Ten candidate values of {0, 0.1, *…*, 0.9} were selected for *dropout*. It can be seen in Figure 3B that the AUPR of our model is stable when *dropout* varies from 0 to 0.8. Thus, we set *dropout = 0.5* for our model.

## 4 Case studies

To further demonstrate the performance of the MKFGCNII model in predicting the potential associations between miRNAs and specific diseases, Esophageal neoplasms, Lung Neoplasms, and Pancreatic Neoplasms were selected for verification. Specifically, we firstly deleted the edges between disease-specific nodes and all miRNAs from the miRNA-disease heterogeneous graph. Then we took the remaining edges containing miRNA nodes and disease nodes as the training set, and the deleted edges were taken as test set. Finally, we sorted the results of the test set and verified it by the dbDEMC (Yang et al. (2010)) and miRCancer (Xie et al. (2013)) datasets. We used dbDEMC as the first verification database, and when a predictive association were not found in the dbDEMC database, we would confirm it in the miRCancer databases. When a predictive association was not validated in both datasets above, we denoted it as *Unconfirmed* in Tables 5–7. So there is only one database will be provided in the Evidence column of Tables 5–7, although we used the two datasets above for validation. In addition, case studies of the full dataset are placed in the supporting materials.

**TABLE 5:** Top 50 miRNAs related to esophageal neoplasms predicted by MKFGCNII.

Rank	miRNA	Evidence	Rank	miRNA	Evidence
1	hsa-mir-375	dbDEMC	26	hsa-mir-200b	miRCancer
2	hsa-mir-200c	dbDEMC	27	hsa-mir-663	dbDEMC
3	hsa-mir-31	dbDEMC	28	hsa-mir-95	dbDEMC
4	hsa-mir-7	dbDEMC	29	hsa-mir-338	dbDEMC
5	hsa-let-7a	miRCancer	30	hsa-mir-9	dbDEMC
6	hsa-mir-21	dbDEMC	31	hsa-mir-133b	dbDEMC
7	hsa-mir-1	dbDEMC	32	hsa-mir-520c	dbDEMC
8	hsa-mir-196a	dbDEMC	33	hsa-mir-126	dbDEMC
9	hsa-mir-218	dbDEMC	34	hsa-mir-203	dbDEMC
10	hsa-mir-142	Unconfirmed	35	hsa-mir-152	dbDEMC
11	hsa-mir-145	dbDEMC	36	hsa-mir-199b	dbDEMC
12	hsa-mir-200a	dbDEMC	37	hsa-mir-222	dbDEMC
13	hsa-mir-521	dbDEMC	38	hsa-mir-494	dbDEMC
14	hsa-mir-107	dbDEMC	39	hsa-mir-561	dbDEMC
15	hsa-mir-486	dbDEMC	40	hsa-mir-223	miRCancer
16	hsa-mir-10b	dbDEMC	41	hsa-mir-22	dbDEMC
17	hsa-mir-18b	dbDEMC	42	hsa-mir-27b	dbDEMC
18	hsa-let-7g	miRCancer	43	hsa-mir-216b	miRCancer
19	hsa-mir-370	dbDEMC	44	hsa-mir-26b	dbDEMC
20	hsa-mir-497	dbDEMC	45	hsa-mir-299	Unconfirmed
21	hsa-mir-16	dbDEMC	46	hsa-mir-18a	dbDEMC
22	hsa-mir-151	dbDEMC	47	hsa-mir-127	dbDEMC
23	hsa-mir-211	dbDEMC	48	hsa-mir-372	dbDEMC
24	hsa-mir-212	dbDEMC	49	hsa-mir-146a	dbDEMC
25	hsa-mir-140	dbDEMC	50	hsa-mir-451a	dbDEMC

### 4.1 Esophageal neoplasms

Esophageal neoplasms is a common type of digestive tract neoplasms with high malignancy and poor prognosis. Five-year survival for malignant esophageal neoplasms is only about 13 ∼18%, even with advanced treatment (Milano and Krishnadath (2008)). The pathogenesis of esophageal tumors is diverse, and it is normally believed to be the result of environment-genetic-gene interaction. But there is no unified and exact conclusion yet. Therefore, further research on the pathogenesis of esophageal tumors is of great significance for its early screening, diagnosis, prevention and prognosis. From Table 5, we can find that a total of 48 of the top 50 miRNAs related to esophageal neoplasms were confirmed in the dbDEMC and miRCancer datasets. For the remaining two miRNAs, we can find their variants associated with esophageal neoplasms in the dbDEMC and miRCancer database. Specifically, the 10th ranked miRNA, hsa-mir-142, its variants hsa-mir-142-3p and hsa-mir-142-5p were found to be associated with esophageal neoplasms in the dbDEMC and miRCancer database. The 45th ranked miRNA, hsa-mir-299, its variants hsa-mir-299-3p and hsa-mir-299-5p were also found to be associated with esophageal neoplasms in the dbDEMC and miRCancer database.

### 4.2 Lung neoplasms

Lung neoplasms is one of the common malignant tumors which occurred in 2.2 million people and resulted in 1.8 million deaths in 2020 (Sung et al. (2021)). In most countries the 5-year survival rate is less than 20%. As miRNAs take part in development, cell proliferation and apoptosis, their deregulation has been concerned with cancer initiation and progression, implying that miRNAs possibly act as neoplasms suppressor genes or oncogenes in various types of lung cancers (Lynam-Lennon et al. (2009)). For excample, (Nadal et al. (2014)), found that miR-370 was upregulated in patients with recurrent tumors, resulting in poor survival in patients with lung adenocarcinoma. Table 6 shows 47 of the top 50 miRNAs related to lung neoplasms in the prediction results of our model. Although 3 miRNAs: hsa-mir-151, hsa-mir-499a and hsa-mir-378a were not validated, their variants, hsa-mir-151-3p, hsa-mir-151-5p, hsa-mir-499a-3p, hsa-mir-499a-5p, hsa-mir-378a-3p, hsa-mir-378a-5p were found to be associated with lung neoplasms by searching the dbDEMC and miRCancer database.

**TABLE 6:** Top 50 miRNAs related to lung neoplasms predicted by MKFGCNII.

Rank	miRNA	Evidence	Rank	miRNA	Evidence
1	hsa-mir-34a	dbDEMC	26	hsa-mir-130a	dbDEMC
2	hsa-mir-486	dbDEMC	27	hsa-mir-487a	dbDEMC
3	hsa-mir-125b	dbDEMC	28	hsa-mir-151	Unconfirmed
4	hsa-mir-93	dbDEMC	29	hsa-mir-7	dbDEMC
5	hsa-mir-155	dbDEMC	30	hsa-mir-199a	dbDEMC
6	hsa-mir-30e	dbDEMC	31	hsa-mir-497	dbDEMC
7	hsa-mir-100	dbDEMC	32	hsa-mir-708	dbDEMC
8	hsa-mir-27b	dbDEMC	33	hsa-mir-30d	dbDEMC
9	hsa-mir-145	dbDEMC	34	hsa-mir-125a	dbDEMC
10	hsa-mir-1	dbDEMC	35	hsa-mir-200b	dbDEMC
11	hsa-let-7g	dbDEMC	36	hsa-mir-658	dbDEMC
12	hsa-mir-16	dbDEMC	37	hsa-mir-488	dbDEMC
13	hsa-mir-424	dbDEMC	38	hsa-mir-135b	dbDEMC
14	hsa-mir-205	dbDEMC	39	hsa-mir-223	dbDEMC
15	hsa-let-7b	dbDEMC	40	hsa-mir-499a	Unconfirmed
16	hsa-mir-21	dbDEMC	41	hsa-mir-144	dbDEMC
17	hsa-mir-196a	dbDEMC	42	hsa-mir-135a	dbDEMC
18	hsa-mir-520d	dbDEMC	43	hsa-mir-15a	dbDEMC
19	hsa-mir-193b	dbDEMC	44	hsa-mir-451a	dbDEMC
20	hsa-mir-181a	dbDEMC	45	hsa-mir-20b	dbDEMC
21	hsa-let-7d	dbDEMC	46	hsa-mir-378a	Unconfirmed
22	hsa-mir-186	dbDEMC	47	hsa-mir-30a	dbDEMC
23	hsa-mir-668	dbDEMC	48	hsa-mir-17	dbDEMC
24	hsa-mir-27a	dbDEMC	49	hsa-mir-34c	dbDEMC
25	hsa-mir-148a	dbDEMC	50	hsa-mir-218	dbDEMC

### 4.3 Pancreatic neoplasms

There are many types of pancreatic tumors, which are difficult to diagnose. Although the incidence rate is low, it has a high degree of malignancy, poor prognosis and short survival time for patients. According to statistics, pancreatic cancer ranks seventh in male malignant tumor incidence, 11th in females, and sixth in malignant tumor-related mortality in China. Researches in the past 2 decades have shown that miRNA and pancreatic tumors are associated. For example, (Rawat et al. (2019)), described the roles of miRNA’s in pancreatic cancer which included diagnosis, prognosis and therapeutic intervention. Thus, we chose pancreatic neoplasms as the third case study for the MKFGCNII model. Table 7 shows that 46 of the top 50 miRNAs associated with pancreatic neoplasms were confirmed in the prediction results of our model. The remaining four miRNAs which have not been verified are hsa-mir-486, hsa-mir-520d, hsa-mir-499a and hsa-mir-378a. But the ariants hsa-mir-486-3p and hsa-mir-486-5p of hsa-mir-486 were found to be associated with pancreatic neoplasms in the dbDEMC database. The same situation also appeared for hsa-mir-520d, hsa-mir-499a and hsa-mir-378a. That is, both -3p and -5p variants of these miRNAs are associated with pancreatic neoplasms in the dbDEMC database.

**TABLE 7:** Top 50 miRNAs related to pancreatic neoplasms predicted by MKFGCNII.

Rank	miRNA	Evidence	Rank	miRNA	Evidence
1	hsa-mir-34a	dbDEMC	26	hsa-mir-130a	dbDEMC
2	hsa-mir-486	Unconfirmed	27	hsa-mir-487a	dbDEMC
3	hsa-mir-125b	dbDEMC	28	hsa-mir-151	dbDEMC
4	hsa-mir-93	dbDEMC	29	hsa-mir-7	dbDEMC
5	hsa-mir-155	dbDEMC	30	hsa-mir-199a	dbDEMC
6	hsa-mir-30e	dbDEMC	31	hsa-mir-497	dbDEMC
7	hsa-mir-100	dbDEMC	32	hsa-mir-708	dbDEMC
8	hsa-mir-27b	dbDEMC	33	hsa-mir-30d	dbDEMC
9	hsa-mir-145	dbDEMC	34	hsa-mir-125a	dbDEMC
10	hsa-mir-1	dbDEMC	35	hsa-mir-200b	dbDEMC
11	hsa-let-7g	dbDEMC	36	hsa-mir-658	dbDEMC
12	hsa-mir-16	dbDEMC	37	hsa-mir-488	dbDEMC
13	hsa-mir-424	dbDEMC	38	hsa-mir-135b	dbDEMC
14	hsa-mir-205	dbDEMC	39	hsa-mir-223	dbDEMC
15	hsa-let-7b	dbDEMC	40	hsa-mir-499a	Unconfirmed
16	hsa-mir-21	dbDEMC	41	hsa-mir-144	dbDEMC
17	hsa-mir-196a	dbDEMC	42	hsa-mir-135a	dbDEMC
18	hsa-mir-520d	Unconfirmed	43	hsa-mir-15a	dbDEMC
19	hsa-mir-193b	dbDEMC	44	hsa-mir-451a	dbDEMC
20	hsa-mir-181a	dbDEMC	45	hsa-mir-20b	dbDEMC
21	hsa-let-7d	dbDEMC	46	hsa-mir-378a	Unconfirmed
22	hsa-mir-186	dbDEMC	47	hsa-mir-30a	dbDEMC
23	hsa-mir-668	dbDEMC	48	hsa-mir-17	dbDEMC
24	hsa-mir-27a	dbDEMC	49	hsa-mir-34c	miRCancer
25	hsa-mir-148a	dbDEMC	50	hsa-mir-218	dbDEMC

## 5 Discussion and conclusion

Since the first miRNA was discovered by (Lee R. et al. (1993)) in 1993, thousands of miRNAs have been identified in humans, and more and more studies show that it plays a critical role in the generation and development of human diseases. In the past 2 decades, a large amount of miRNA-related data have been generated through various biological experiments. On this basis, the association of miRNA-disease databases, such as dbDEMC and miRCancer, has also been established. Using these databases and computational methods can not only reduce the cost and cycle time of traditional biological experiments, but also lead researchers to research into certain miRNA-disease associations. In this paper, we proposed an miRNA–disease association prediction method based on multiple kernel fusion on GCNII to predict the potential associations between miRNAs and diseases, called the MKFGCNII model. The model applied GCNII module, which can solve the over-smoothing problem to a large extent, to extract embedding information layer by layer. Then it generated miRNA kernal and disease kernal for each layer and fused all this kernel matrices based on a weighting method. Finally, Dual Graph Regularized Least Squares (DLapRLS) was used to predict the predict miRNA–disease associations. Based on the GCNII model’s ability to solve over-smoothing, we superimposed the hidden layer of the model to 16 layers, adding two graph attention layers before the hidden layer and one fully connection layer after the hidden layer. All these layers provided DLapRLS with enough kernels for prediction, thereby improving the performance of the MKFGCNII model.

However, the MKFGCNII model still has some disadvantages, which will be investigated and discussed in the future. The correlation matrix between miRNAs and diseases is sparse, which causes the model predictions to be biased towards negative class samples. Second, the dimension of the hidden layer is fixed which should be further studied. In addition, the current miRNA-disease association analyses can be divided into two categories: single-relationship analysis and multi-relationship analysis. Single-relationship analysis, which is to analyze whether there is an association between miRNA and disease, obtains high accuracy yet fails in predicting the category of the association; while multi-relationship analysis, which is to analyze which kind of association exists between miRNA and disease, is able to predict the category of the association but with low accuracy. Neither is perfect. Therefore, we believe that being able to predict the category of miRNA-disease association with high accuracy will be one of the future development directions. We will work on this direction in future research.

## Data Availability

The original contributions presented in the study are included in the article/supplementary files, further inquiries can be directed to the corresponding author.
